# Analysis of Conventional Ultrasound and Contrast-Enhanced Ultrasound Features of Pseudoangiomatous Stromal Hyperplasia

**DOI:** 10.1155/tbj/6070736

**Published:** 2025-07-10

**Authors:** Hui Li, Qinghua Niu, Chao Jia, Gaoxiang Fan, Long Liu, Gang Li, Penglin Zou, Rong Wu, Lianfang Du, Jing Wang, Qiusheng Shi

**Affiliations:** ^1^Department of Ultrasound, Huashan Hospital, Fudan University, Shanghai, China; ^2^Department of Ultrasound, Shanghai General Hospital, Shanghai Jiao Tong University School of Medicine, Shanghai, China; ^3^Department of Pathology, Shanghai General Hospital, Shanghai Jiao Tong University School of Medicine, Shanghai, China

**Keywords:** breast, contrast-enhanced ultrasound, conventional ultrasound, pseudoangiomatous stromal hyperplasia

## Abstract

**Purpose:** To investigate the conventional ultrasound and contrast-enhanced ultrasound (CEUS) imaging features of pseudoangiomatous stromal hyperplasia (PASH).

**Methods:** Retrospective analysis of clinical and imaging data of 29 patients diagnosed with PASH from June 2014 to June 2023.

**Results:** The median age of the patients was 39 years. Linear/cystic hypoechoic areas could be detected within the lesion in 12 cases (41.4%), and in 17 cases, the lesions had extensive conventional ultrasound findings with no significant features. The ultrasound-measured lesion diameters were smaller than those measured in surgically resected lesions, and the statistical difference was highly significant (*p* < 0.01). Fifteen cases underwent CEUS examination, with 7 lesions (46.7%) demonstrating uniform enhancement and 8 lesions (53.3%) exhibiting nonuniform enhancement. Within the enhanced regions, perfusion defects were observed, all of which were of the patchy type. The areas of patchy perfusion defects corresponded to the linear/cystic hypoechoic regions observed in the conventional sonographic images of the lesions. The use of CEUS provided additional diagnostic clarity compared with conventional ultrasound. Specifically, the specificity for identifying PASH lesions increased from 35.7% with conventional ultrasound to 64.3% with CEUS, highlighting the value of CEUS in enhancing the diagnostic accuracy for PASH lesions.

**Conclusion:** This study suggests that linear/cystic hypoechoic areas on sonography may serve as crucial clues for the ultrasound diagnosis of PASH. The presence of diffuse patchy perfusion defects in CEUS contributes to the accurate diagnosis of PASH.

## 1. Introduction

Pseudoangiomatous stromal hyperplasia (PASH) is a rare benign stromal proliferative lesion of the breast, first described by Vuitch et al. [1] in 1986. The histological feature is the presence of slit-like channels within the myofibroblast-lined mesenchyme, resembling vascular channels, which can be misdiagnosed as low-grade angiosarcoma and phyllodes tumors [2]. PASH has a wide range of clinical presentations, from incidental microscopic findings accompanying other breast lesions to rapidly growing palpable masses [3]. Most often diagnosed incidentally on histological examination of other benign or malignant lesions. Polger et al. [4] and Yoon et al. [5] reported that incidental microscopic PASH could be found in 23% of breast biopsy specimens. The exact etiology and pathogenesis of PASH are still unclear, and it is currently mainly considered to be a hormone-responsive disease, most commonly seen in premenopausal or perimenopausal women [6, 7], and individual case reports have been reported in children, adolescents, and men [8–10].

Radiologically, PASH has no specific imaging features [11]. The ultrasound features of PASH are nonspecific, most often presenting as a hypoechoic, oval, confined mass [12], which is difficult to differentiate from fibroadenoma. The application value of contrast-enhanced ultrasound (CEUS) in multiple organ neoplastic lesions has been recognized [13]. CEUS is successfully utilized to diagnose a range of pathologies and holds clinical relevance in the management of breast cancer. It can provide the possibility to predict molecular subtypes and assess the efficacy of neoadjuvant chemotherapy, and it can also aid in the detection and characterization of lymph nodes [14, 15]. CEUS is recognized as a high-performance, practical, and noninvasive imaging technique, characterized by its lack of ionizing radiation, broad accessibility, and straightforward implementation. It has proven to be a valuable addition to breast ultrasound [16]. CEUS is able to show the microcirculatory perfusion within the tumor, providing additional diagnostic information for qualitative and quantitative analysis of the lesion [17] and thus can have a high accuracy in distinguishing benign and malignant lesions. Some benign lesions have specific characteristics in CEUS. For example, breast cysts generally show no enhancement in the lesion after contrast. The vast majority of breast fibroadenoma showed rapid regression after contrast. But there is no case or series report on the CEUS sonographic characteristics of PASH. In this study, 29 patients with PASH in our hospital during the past ten years were analyzed by conventional ultrasound and CEUS in order to find the sonographic clues for PASH diagnosis.

## 2. Materials and Methods

### 2.1. Patient

**Figure 1:**
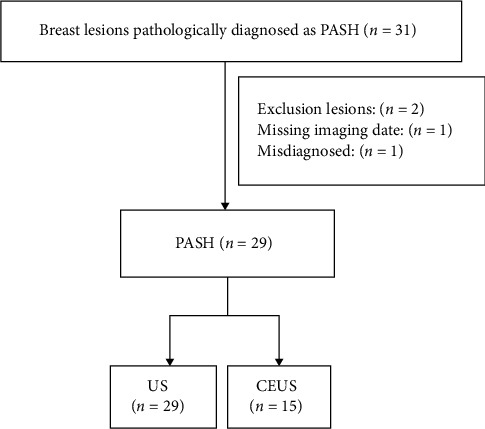
Study population flowchart.

The clinical data of 31 patients diagnosed with PASH from June 2014 to June 2023 were retrospectively reviewed. Among the 31 patients, 1 case of osteosarcoma metastasis to the anterior chest wall was incidentally found in the resected breast tissue and was excluded from this study due to missing imaging data. One case of phyllodes tumor was excluded from this study because CNB was misdiagnosed as PASH. Twenty-nine patients finally participated in the study. The medical records of these patients were reviewed, including demographic data, clinical characteristics, conventional ultrasound and CEUS image data, and pathological results (Figure 1).

### 2.2. Imaging Techniques

Scanning was performed using color Doppler devices (Aplio 500 and Aplio i900; Canon Medical Systems, Otawara, Japan) with 14-5L and 14-8L probes with frequencies of 5–14 and 8–14 MHz, respectively. Before the examination, the patient was placed in the supine position with both upper limbs elevated, both breasts were fully exposed, and the patient breathed calmly. A comprehensive ultrasound scan of both breasts was performed to detect lesions and the images were stored. Long-axis and short-axis views of the lesion were shown on conventional images.

CEUS was performed immediately after routine ultrasound examination. The maximum diameter section of the lesion was selected to fix the probe, and the dual-amplitude mode was used to accurately locate the lesion. CEUS was conducted utilizing the contrast harmonic imaging mode, with the mechanical index meticulously set at 0.07 to ensure patient safety and image quality. The contrast agent was SonoVue (Bracco, Italy), which was prepared according to the manufacturer's recommendation. A total of 4.8 mL of contrast agent was injected into the elbow vein, followed by 5 mL of normal saline to flush the tube. Following contrast administration, continuous observation was performed for 1 min. Video recording was started simultaneously with the contrast agent injection. The ultrasound procedures were performed by two doctors with more than 10 years of experience in CEUS.

### 2.3. Image Analysis

Conventional ultrasonic image analysis parameters include the lesion size; shape (round/oval or irregular); margin (circumscribed or noncircumscribed); internal echo (homogeneous or presence of linear/cystic hypoechoic areas); and calcification (yes or none). Lesions were categorized into subcategories 4a, 4b, and 4c based on the ultrasound images using the 5th edition of ACR BI-RADS criteria [18]. The conventional ultrasound images were analyzed and evaluated by two senior sonographers with 20 years of experience.

CEUS image analysis parameters included: enhanced time (earlier, the same, or later); enhanced shape (regular or irregular); enhanced margin (well defined or poor defined); enhanced order (centripetal or not centripetal); contrast agent distribution (homogeneous or heterogeneous); perfusion defect (presence or absence); enhanced intensity at peak time (hyperenhancement or iso-/hypoenhancement); whether the enhanced range is increased compared with conventional ultrasound; and contrast agent retention in the venous phase (presence or absence). Perfusion defects were further divided into regional or diffuse patchy. Regional defects were defined as nonperfusion areas within the lesions and patchy defects were punctate or slit-filling defect in the lesions [19]. The CEUS images were analyzed and evaluated by two senior sonographers with 10 years of experience in CEUS. All images were reclassified using a 5-point scoring system developed by Xiao et al. [20] .

### 2.4. Pathological Findings

All 29 patients were histologically confirmed after surgical resection, and all pathological data were interpreted by two pathologists with more than 10 years of experience in diagnosing breast disease.

### 2.5. Statistical Analysis

SPSS 26.0 software was used to analyze and process the data. All continuous variables were expressed as the median (interquartile range) depending on their distribution. Count data were expressed as cases (%). Comparisons between groups were analyzed using nonparametric tests, with *p* < 0.05 being considered a significant difference and *p* < 0.01 being considered a highly significant difference.

## 3. Results

### 3.1. Characteristics of Patients

All 29 patients were female and the median age was 39 years. The median size of surgical pathological lesions was 25 mm. Nineteen patients (65.5%) had lesions detected by physical examination screening without complaints of discomfort, but all of them were able to palpate the mass without tenderness during physical examination. Ten patients (34.5%) had lesions detected by subjective palpation of the mass, and one of them (10%) had tenderness on physical examination. Details are shown in Table 1.

### 3.2. Sonographic Representation

**Figure 2:**
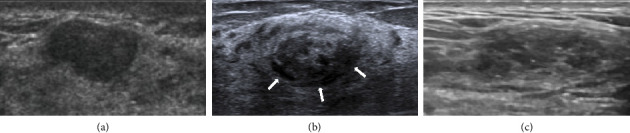
(a) Ultrasound presentation reveals an elliptical, well-defined hypoechoic mass with clear boundaries, (b) cystic hypoechoic areas could be detected (“↑”), and (c) punctate hyperechogenicity was seen within the lesion.

All 29 patients underwent conventional ultrasound examinations. The median size of the lesions measured by ultrasound was 15 mm. Among the 29 lesions, 21 (72.4%) exhibited a round/oval shape (Figure 2(a)), while 8 (27.6%) were irregular. The margins of 25 lesions (86.2%) were clearly defined (Figure 2(a)), while 4 (13.8%) had indistinct margins. Internal echogenicity was uniform in 17 cases (58.6%) (Figure 2(a)) and demonstrated linear/cystic hypoechoic areas in 12 cases (41.4%) (Figure 2(b)). Calcifications were absent in 20 cases (69.0%) and present in 9 cases (31.0%) (Figure 2(b)). The sonographic presentation is detailed in Table 2.

**Figure 3:**
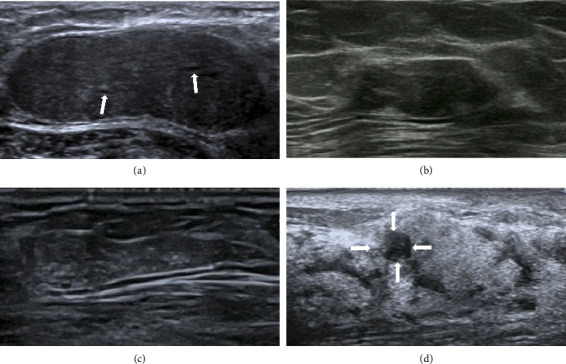
(a) Conventional ultrasound suggests BI-RADS 3, with clear margins, regular morphology, and linear hypoechoic structures within the lesion (”↑“); (b) conventional ultrasound suggests BI-RADS 4a, with indistinct margins and somewhat irregular morphology; (c) conventional ultrasound indicates BI-RADS 4b, with indistinct margins, irregular morphology, and internal punctate hyperechoic foci; and (d) conventional ultrasound indicates BI-RADS 4c, with indistinct margins, irregular morphology, and internal punctate hyperechoic foci, and the lesion demonstrates nonmass-like changes. It solely suggests a hypoechoic lesion (“↑”) mixed with the breast parenchyma.

Among the 29 lesions, 16 (56.7%) were classified as BI-RADS 3 (Figure 3(a)), 11 (36.7%) as BI-RADS 4a (Figure 3(b)), 1 (3.3%) as BI-RADS 4b (Figure 3(c)), and 1 (3.3%) as BI-RADS 4c (Figure 3(d)). Lesions categorized as BI-RADS 4b exhibited irregular shape, indistinct margins, heterogeneous internal echogenicity, and the presence of echogenic foci (Figure 3(c)). Lesions classified as BI-RADS 4c displayed irregular shape, indistinct margins, heterogeneous internal echogenicity, the presence of echogenic foci, and demonstrated nonmass-like changes (Figure 3(d)).

Seven lesions appeared as hypoechoic lesions, which could not be distinguished from the breast parenchyma, resulting in a significant difference between conventional ultrasound-measured lesion size and postoperative pathological size (Figure 3(d)). Details are shown in Table 3. The ultrasound-measured lesion meridians were smaller than the surgically excised lesion measurements, and the statistical difference was highly significant (*z* = −2.716, *p*=0.007).

**Figure 4:**
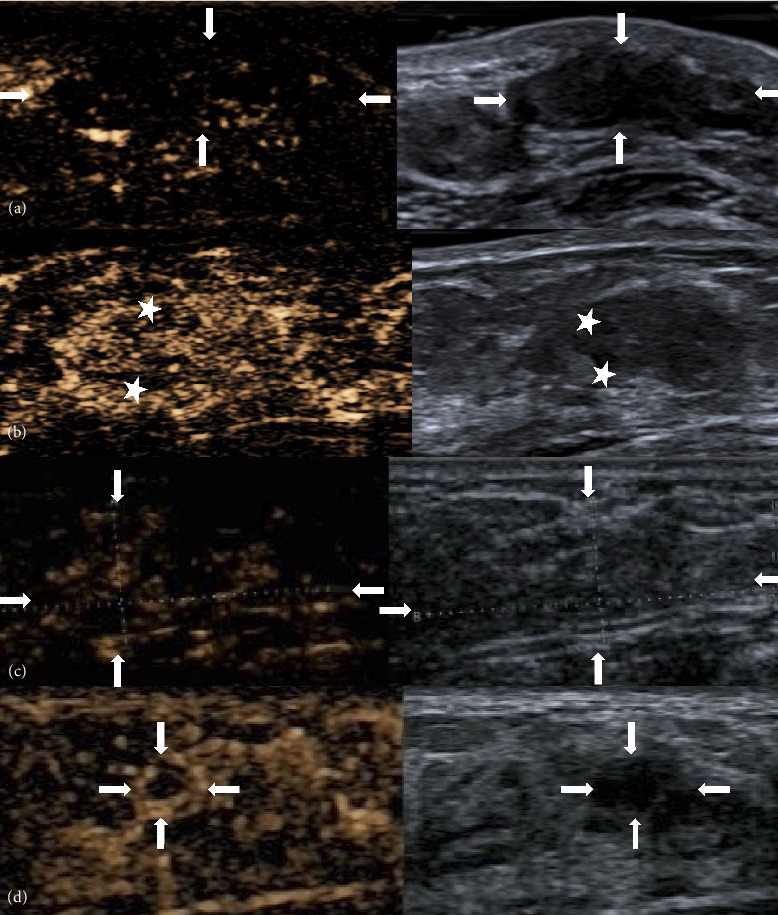
(a) CEUS demonstrates the lesion as having low enhancement, with uneven perfusion showing a low perfusion pattern. After enhancement, the lesion exhibits clear boundaries and regular morphology (as indicated by “↑”); (b) CEUS reveals uneven enhancement with perfusion defects, presenting as patchy perfusion defects (depicted by “★”), the defective perfusion areas correspond to the cystic hypoechoic regions (indicated by “★”), and after enhancement, the lesion demonstrates clear boundaries and regular morphology; and (c) conventional ultrasound indicates BI-RADS 4b, while CEUS reveals low enhancement with internal perfusion defects. CEUS findings suggest BI-RADS 4a (as indicated by “↑”). (d) Conventional ultrasound suggests BI-RADS 4c, while CEUS reveals peripheral high enhancement, central low enhancement, uneven contrast agent perfusion, clear boundaries, and regular morphology. CEUS findings suggest BI-RADS 3 (as indicated by “↑”).

Fifteen patients (51.7%) underwent CEUS examinations. The median size of the lesions measured by CEUS was 12.2 mm (8.2–21 mm). On CEUS images, 7 of the 15 lesions (46.7%) demonstrated uniform enhancement, while 8 lesions (53.3%) exhibited nonuniform enhancement (Figure 4(a)). Among the 15 lesions, 8 (53.3%) showed perfusion defects, while 7 (46.7%) did not. In the 8 lesions with perfusion defects, the defect patterns were consistently patchy, corresponding to the areas of linear/cystic hypoechoic regions identified in the routine sonographic images (Figure 4(b)). See Table 4 for detailed imaging characteristics.

In the 15 cases where both US and CEUS were performed, CEUS downgraded lesions that were initially categorized as BI-RADS 4 by US to BI-RADS 3 in 8 instances. One lesion initially classified as BI-RADS 4b on routine ultrasound were downgraded to BI-RADS 4a on CEUS (Figure 4(c)), and one lesion initially classified as BI-RADS 4c on routine ultrasound were downgraded to BI-RADS 3 on CEUS (Figure 4(d)). CEUS improved the specificity from 35.7% to 64.3% (Table 5), thereby allowing patients to avoid unnecessary percutaneous biopsies.

### 3.3. Pathological Findings

**Figure 5:**
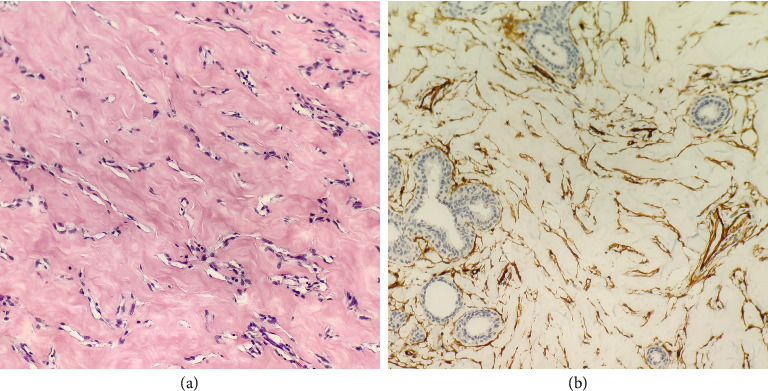
(a) Within the dense collagen matrix, there are intricate fissure-like gaps of complex apposition. These gaps are lined by myofibroblasts rather than endothelial cells and do not contain red blood cells (hematoxylin and eosin, magnification × 200). (b) The lining cells are positive for CD 34 (immunohistochemical staining, magnification × 200).

After surgical excision, tissue pathology confirmed diagnoses in all 29 patients. Diagnoses included PASH (19/29), PASH combined with fibroadenoma (6/29), PASH combined with breast cyst (2/29), PASH combined with atypical ductal hyperplasia (1/29), and PASH combined with complex sclerosing lesions, fibroadenoma, intraductal papilloma, and atypical ductal hyperplasia (1/29). The final pathological report for PASH is detailed in Table 6. Among the cases, 10 underwent immunohistochemical examination, all of which showed positive CD34 staining consistent with myofibroblastic differentiation (Figures 5(a) and 5(b)).

## 4. Discussion

PASH is a benign interstitial proliferative lesion of the breast, which is composed of slit-like channels similar to vascular spaces [1]. It can present as a palpable breast mass that is indistinguishable from other benign breast masses. PASH has a wide range of radiological manifestations, and it is difficult to diagnose PASH only by radiological manifestations at present. Hargaden et al. [6] reported that 69% of the patients had no abnormalities detected on mammography even though PASH was the main pathological diagnosis. When visible on mammography, PASH is most commonly seen as a round or oval mass with well-defined or partially well-defined boundaries and no calcification [21], and some may show focal asymmetry [6]. There are fewer studies on the MRI manifestations of PASH, which may present as showing low or equal signal on T1-weighted images and high signal on T2-weighted images [22]. Nia et al. [23] reported that the MRI manifestations of PASH were not related to the percentage of PASH in pathological specimens.

The lesions in all 29 patients in our dataset were detectable on ultrasound. The ultrasound features of PASH are nonspecific. Ferreira et al. [24] and Jung and Kim [25] reported that the most common ultrasound feature of PASH was a round or oval hypoechoic mass in a parallel orientation without posterior echogenic enhancement. The most common ultrasound feature of PASH was a round or oval hypoechoic mass in a parallel orientation without posterior echogenic enhancement. In this study, 10 cases (34.5%) manifested as round or oval, well-defined, hypoechoic masses with parallel orientation. Abdull Gaffar [26] and Drinka et al. [27] reported that in some cases, lesions could exhibit heterogeneous internal echogenicity, irregular or indistinct borders, posterior acoustic enhancement, and visible cystic components. In our study, 41.4% of the lesions showed linear or cystic hypoechoic patterns within (see Figure 2(b)), a significantly higher proportion compared with the findings reported by Abdull Gaffar et al. [26] and Drinka et al. [27]. Virk et al. [22] reported that the histological hallmark of PASH is the presence of open slit-like spaces within a dense collagenous matrix. These spaces are lined by a discontinuous layer of flattened, spindle-shaped myofibroblastic cells. The unevenness created by these spaces and their lining myofibroblasts may manifest as fissure-like hypoechoic or linear structures on sonographic imaging. We hypothesize that the linear or cystic hypoechoic patterns observed on ultrasound correspond to histopathological alterations in the form of slit-like channels. Therefore, we believe that the presence of linear or cystic hypoechoic areas might serve as crucial sonographic indicators suggestive of PASH. In this study, calcifications were observed in ultrasound images in nine cases, a feature not previously reported in the literature. Disturbances in calcium metabolism, proliferation of ductal epithelial cells, inflammatory reactions, and capillary hemorrhage could contribute to the appearance of calcifications within the breast [28]. Ultrasound measurements of lesions in this study were significantly smaller than the measurements postsurgical excision, with a highly significant statistical difference (*p*=0.007). In 7 cases, there was a significant discrepancy between the conventional ultrasound measurements of lesion size and the postoperative pathological size (detailed in Table 3). Upon analysis, the reason for this disparity was identified: all 7 cases presented as nonmass-like lesions, appearing hypoechoic on ultrasound, and their boundaries were indistinct due to intermingling with the surrounding breast parenchyma, leading to measurement errors in lesion sizing. Nonmass-like lesions in the breast, characterized by a complex appearance on ultrasound, can result in unclear borders or morphologies. Therefore, when lesions exhibit a nonmass-like appearance, caution is advised in the assessment to minimize underestimation of lesion size during measurements.

CEUS is a pure blood pool imaging technique that allows real-time and continuous observation of perfusion changes and the microvascular structure of lesions, aiding in the differentiation of benign and malignant breast lesions [29]. There have been no reported CEUS features specifically for PASH. Studies by Du et al. [30] suggest that the presence of perfusion defects is a characteristic of malignant breast lesions. In further research, Xia et al. [19] classified perfusion defects into regional and patchy types, asserting that regional perfusion defects are associated with malignant breast lesions, while patchy perfusion defects are more common in benign lesions. However, the mechanism behind diffuse patchy perfusion defects remains unknown. In our study cohort, among the 15 lesions examined with CEUS, 8 lesions (53.3%) exhibited perfusion defects. The perfusion defects in these 8 lesions were of the patchy type, and the areas of diffuse patchy perfusion defects corresponded to the linear/cystic hypoechoic regions observed in the conventional sonographic images of the lesions. We hypothesize that the histopathological alterations in PASH tissue, characterized by slit-like channels, may be responsible for the occurrence of diffuse patchy perfusion defects within the lesions. Therefore, we posit that the presence of linear or cystic hypoechoic areas on conventional sonography, consistent with the diffuse patchy perfusion defect areas on CEUS, could be a significant sonographic clue further suggesting the presence of PASH.

In immunohistochemistry, spindle cells lining the stroma show positive expression for the myofibroblastic markers CD34, smooth muscle actin (SMA), vimentin, desmin, Bcl2, and progesterone receptor. They exhibit negative staining for von Willebrand factor (VIII), CD31, S100, and cytokeratin [26]. In this study, immunohistochemical examination was performed on 10 cases, and CD34 displayed consistent positive expression, aligning with previous reports such as Damiani et al. [31], which is consistent with the immunophenotype of myofibroblastic cells. Bowman et al. [2] reported that histologically, PASH may be challenging to differentiate from phyllodes tumors and angiosarcomas. In this dataset, one case diagnosed as PASH on core needle biopsy was later identified as a benign phyllodes tumor after subsequent surgical excision. PASH lesions typically reside in the interlobular or intralobular stroma, displaying low mitotic counts and absent to mild nuclear atypia. These are specific features distinguishing PASH from low-grade vascular sarcomas or phyllodes tumors [12].

While previous studies have explored the ultrasound features of PASH, our work introduces a comprehensive multimodal imaging approach that includes both conventional ultrasound and CEUS. This dual-modality analysis allows for a more detailed characterization of PASH lesions. Specifically, we identified linear/cystic hypoechoic areas and diffuse patchy perfusion defects as key diagnostic features, which have not been thoroughly explored in prior research. Moreover, our study demonstrates a significant improvement in diagnostic specificity when CEUS is used in conjunction with conventional ultrasound, highlighting the clinical utility of this combined approach. These findings not only enhance the diagnostic accuracy for PASH but also pave the way for future research into advanced imaging techniques and predictive modeling.

Our study reports that some PASH lesions were found in combination with other conditions, including fibroadenoma, breast cyst, atypical ductal hyperplasia, and complex sclerosing lesions. These combinations can complicate the interpretation of the results, as each type of lesion may have different characteristics that influence imaging findings and pathological results. For instance, fibroadenomas can present with well-defined borders and homogeneous echotexture, which might overlap with the appearance of PASH lesions on ultrasound. Similarly, breast cysts can exhibit anechoic or hypoechoic regions, potentially masking the linear/cystic hypoechoic areas characteristic of PASH. To mitigate this complexity, we carefully analyzed the imaging features in the context of the overall clinical presentation and pathological findings. We focused on identifying distinct patterns, such as the presence of linear/cystic hypoechoic areas and specific enhancement patterns on CEUS, which could be associated with PASH even in the presence of mixed pathology. We recognize that the coexistence of these lesions introduces variability and potential biases in our analysis. Therefore, we have highlighted this as a significant limitation in our study and emphasized the need for further research with larger, more homogeneous cohorts to validate our findings.

## 5. Conclusion

This study suggests that linear/cystic anechoic findings within the mass may be an important clue for the diagnosis of PASH by ultrasound. The diffuse patchy defect in CEUS is helpful for the correct diagnosis of PASH.

## Figures and Tables

**Table 1:** Characteristics of patients with PASH.

Characteristic	No. (%) (*N* = 29)
Age	39 (32.0, 36.0)
Lesion size	25 (17.5, 50.0)
Detection	
Health screening image test	19 (65.5%)
Subjective symptom	10 (34.5%)
Incidental finding during evaluation other lesion	0
Subjective symptom	
None	19 (65.5%)
Palpable mass	10 (34.5%)
Tenderness of the lesion	
None	28 (96.6%)
Yes	1 (3.4%)
Palpability of the lesion	
None	0
Yes	29 (100.0%)
Lesion location	
Right breast	12 (41.4%)
Left breast	17 (58.6%)

**Table 2:** Sonographic features on conventional US of PASH.

Characteristic	No. (%) (*N* = 29)
Lesion size (mm)	15 (10.5, 26)
Shape	
Round or oval	21 (72.4%)
Irregular	8 (27.6%)
Margin	
Circumscribed	25 (86.2%)
Not circumscribed	4 (13.8%)
Echo pattern	
Hypoechoic	17 (58.6%)
Hypoechoic linear or cystic	12 (41.4%)
Calcification	
Yes	9 (31.0%)
None	20 (69.0%)
BI-RADS classification	
3	16 (55.2%)
4a	11 (38.0%)
4b	1 (3.4%)
4c	1 (3.4%)

*Note:* Hypoechoic linear or cystic: this term describes a specific type of echo pattern where the lesion appears as dark (hypoechoic) linear structures that contain cystic components.

**Table 3:** Differential lesion characteristics of PASH.

Case	Image size on US (mm)	Image size on CEUS (mm)	Pathological maximum diameter line (mm)
1	5.9	4.4	20.0
2	11.0	12.8	50.0
3	5.7	—	40.0
4	5.5	12.0	45.0
5	11.6	11.6	50.0
6	4.1	6.0	20.0
7	4.8	—	60.0

*Note:* There was a significant difference between the ultrasound measurements of the lesion's longitudinal axis and those of the surgically excised lesion (*z* = −2.716, *p*=0.007).

**Table 4:** Enhancement features on CEUS of PASH.

Characteristic	No. (%) (*N* = 15)
Lesion size (mm)	12.2 (8.2, 21.0)
Enhanced time	
Earlier	6 (40.0%)
Other	9 (60.0%)
Enhanced shape	
Regular	13 (86.7%)
Irregular	2 (13.3%)
Enhanced margin	
Well defined	15 (100.0%)
Poor defined	0
Enhanced order	
Centripetal	11 (73.3%)
Not centripetal	4 (26.7%)
Contrast agent distribution	
Homogeneous	7 (46.7%)
Heterogeneous	8 (53.3%)
Perfusion defect	
Absence	7 (46.7%)
Presence	8 (53.3%)
Enhanced scope	
Enlarged	4 (26.7%)
Other	11 (73.3%)
Contrast agent retention	
Absence	6 (40.0%)
Presence	9 (60.0%)
Enhanced intensity at peak time	
Hyperenhancement	12 (80.0%)
Iso-/hypoenhancement	3 (20.0%)
BI-RADS classification	
3	10 (66.7%)
4a	5 (33.3%)

**Table 5:** The diagnostic efficacy of US and combined CEUS.

	Sensitivity (%)	Specificity (%)	Accuracy (%)	PPV (%)	NPV (%)
US	0.0	35.7	35.7	0.0	100.0
US + CEUS	0.0	64.3	64.3	0.0	100

**Table 6:** Pathological diagnosis of surgical resection.

Pathology	No. (%) (*N* = 29)
PASH	19 (65.5%)
PASH with FA	6 (20.7%)
PASH with breast cyst	2 (6.8%)
PASH with ADH	1 (3.5%)
PASH with CSL, FA, IDP, ADH	1 (3.5%)

Abbreviations: ADH = atypical ductal hyperplasia, CSL = complex sclerosing lesions, FA = fibroadenoma, IDP = intraductal papilloma.

## Data Availability

The data used to support the findings of this study are available on request from the corresponding authors.
