# Corrigendum to “Analysis of Conventional Ultrasound and Contrast-Enhanced Ultrasound Features of Pseudoangiomatous Stromal Hyperplasia”

**DOI:** 10.1155/tbj/9801706

**Published:** 2025-09-19

H. Li, Q. Niu, C. Jia, et al., “Analysis of Conventional Ultrasound and Contrast-Enhanced Ultrasound Features of Pseudoangiomatous Stromal Hyperplasia,” *The Breast Journal* 2025 (2025): 6070736, https://doi.org/10.1155/tbj/6070736.

In the article titled “Analysis of Conventional Ultrasound and Contrast-Enhanced Ultrasound Features of Pseudoangiomatous Stromal Hyperplasia,” there was an error in affiliations 1 and 2 order. The corrected author list and affiliation list should be as follows:

Hui Li^1,2^, Qinghua Niu^1^, Chao Jia^1^, Gaoxiang Fan^1^, Long Liu^1^, Gang Li^1^, Penglin Zou^1^, Rong Wu^1^, Lianfang Du^1^, Jing Wang^3^, and Qiusheng Shi^1^

^1^Department of Ultrasound, Shanghai General Hospital, Shanghai Jiao Tong University School of Medicine, Shanghai, China

^2^Department of Ultrasound, Huashan Hospital, Fudan University, Shanghai, China

^3^Department of Pathology, Shanghai General Hospital, Shanghai Jiao Tong University School of Medicine, Shanghai, China

We apologize for this error.

